# Universal Stress Protein Exhibits a Redox-Dependent Chaperone Function in *Arabidopsis* and Enhances Plant Tolerance to Heat Shock and Oxidative Stress

**DOI:** 10.3389/fpls.2015.01141

**Published:** 2015-12-21

**Authors:** Young Jun Jung, Sarah Mae Boyles Melencion, Eun Seon Lee, Joung Hun Park, Cresilda Vergara Alinapon, Hun Taek Oh, Dae-Jin Yun, Yong Hun Chi, Sang Yeol Lee

**Affiliations:** Division of Applied Life Science (BK21^+^), Plant Molecular Biology and Biotechnology Research Center, Gyeongsang National UniversityJinju, South Korea

**Keywords:** heat shock, high molecular weight (HMW) complex, low molecular weight (LMW) complex, molecular chaperone, oxidative stress, redox status, universal stress protein (USP)

## Abstract

Although a wide range of physiological information on Universal Stress Proteins (USPs) is available from many organisms, their biochemical, and molecular functions remain unidentified. The biochemical function of AtUSP (At3g53990) from *Arabidopsis thaliana* was therefore investigated. Plants over-expressing AtUSP showed a strong resistance to heat shock and oxidative stress, compared with wild-type and *Atusp* knock-out plants, confirming the crucial role of AtUSP in stress tolerance. AtUSP was present in a variety of structures including monomers, dimers, trimers, and oligomeric complexes, and switched in response to external stresses from low molecular weight (LMW) species to high molecular weight (HMW) complexes. AtUSP exhibited a strong chaperone function under stress conditions in particular, and this activity was significantly increased by heat treatment. Chaperone activity of AtUSP was critically regulated by the redox status of cells and accompanied by structural changes to the protein. Over-expression of AtUSP conferred a strong tolerance to heat shock and oxidative stress upon *Arabidopsis*, primarily *via* its chaperone function.

## Introduction

Because plants are sessile organisms, their growth, development, and survival are significantly affected by a variety of external stresses including cold, heat, water deficit, or drought, flooding, high salinity, and strong winds. These stresses can cause production of reactive oxygen species (ROS) containing hydrogen peroxide (H_2_O_2_), superoxide anion ($\cdot {\text{O}}_{2}^{-}$), singlet oxygen (^1^O_2_) and hydroxyl radical (·OH) (Baier and Dietz, 2005; D'Autréaux and Toledano, 2007; Schwarzländer and Finkemeier, 2013). Depending upon the levels of ROS, many downstream signaling systems in cells, such as protein kinases, phosphatases, transcription factors, molecular chaperones, and defense-related proteins, may be activated. ROS thus play dual roles, acting both as useful signaling molecules to sense and activate defense signaling cascades (Møller and Sweetlove, 2010) and as harmful byproducts of aerobic metabolism of plants.

The Universal Stress Protein domain (USP) gene, which encodes a protein containing the 140–160 highly conserved residues of the Universal Stress Protein A domain (USPA, Pfam accession number PF00582), is a representative stress responsive gene. It has been shown to respond to diverse environmental stresses, such as salt, drought, cold, heat, and oxidative stress (Kerk et al., 2003; Ndimba et al., 2005; Persson et al., 2007). The *USP* genes are widely distributed across most living organisms, including bacteria, archaea, fungi, protozoa, plants, and mammals.

The C13.5 protein was first identified from bacteria. Its name was changed to USP to reflect its ability to respond to diverse stresses (Zarembinski et al., 1998; Sousa and McKay, 2001). USPs from *Escherichia coli* have been divided into four classes according to their protein structures and amino acid sequences: Class 1 (UspA, UspC, and UspD), Class 2 (UspG and UspF), and Classes 3 and 4 (two domains of UspE) (Nyström and Neidhardt, 1992; Persson et al., 2007). The bacterial USPs are involved in processes including iron scavenging, oxidative stress resistance, cell adhesion, and cell motility (Nachin et al., 2005). The USP domain in MJ0577 (also known as 1MJH) from *Methanocaldococcus jannaschii* contains an ATP-binding motif and may function biochemically as an ATPase or ATP-binding molecular switch. By contrast, the USP domain in *Haemophilus influenzae* has neither ATP-binding residues nor ATP-binding activity (Sousa and McKay, 2001; Kvint et al., 2003; Persson et al., 2007).

Despite this wide range of physiological information on bacterial USP proteins, the biochemical, and molecular mechanisms of USPs have never been identified. This prompted us to investigate the biochemical functions of these proteins in plants. The genome of *Arabidopsis thaliana* contains 44 proteins homologous to bacterial USPs, based on their protein sequences and structural similarities. Sequence analysis of USPs from *Arabidopsis* suggests these proteins evolved from a 1MJH-like ancestor protein (Kerk et al., 2003).

Two USPs from *Arabidopsis*, AtPHOS32 and AtPHOS34, were phosphorylated in response to microbial elicitation and AtPHOS32 was shown to be a substrate of MAP kinases 3 and 6 (Shinozaki and Yamaguchi-Shinozaki, 2007; Coetzer et al., 2010). In rice, OsUSP1 was shown to activate a cellular downstream signaling cascade in response to ethylene, a gaseous hormone in plant, enabling adaptation of plants to hypoxic conditions (Sauter et al., 2002). Similarly, the USP genes of *Gossypium arboretum, Astragalus sinicus, Solanum pennellii*, and *Salicornia brachiate* are involved in water stress and nodulation, and drought, salt, and osmotic tolerances (Chou et al., 2007; Maqbool et al., 2009; Loukehaich et al., 2012; Udawat et al., 2014).

We examined the biochemical and molecular functions of AtUSP (At3g53990) by analyzing plants over-expressing AtUSP as well as an *Atusp* knock-out mutant, and also recombinant AtUSP. Plants over-expressing AtUSP showed a strong resistance to heat shock and oxidative stress. In particular, we demonstrated that AtUSP exhibited a molecular chaperone function and that chaperone activity was critically regulated in a redox and heat shock-dependent manner, accompanied by structural changes to the protein. The chaperone function of AtUSP thus plays an essential role in protecting plants from heat shock and oxidative stress.

## Materials and methods

### Plants and growth conditions

*A. thaliana* (Columbia ecotype) plants were grown under a 16/8 h light/dark cycle at 22°C and 70% humidity. The T-DNA insertion knock-out mutant line (SALK_146059) was obtained from the Arabidopsis Biological Resource Center (USA). *Arabidopsis* seeds were surface-sterilized and sown either onto solid MS medium containing 0.25% phytagel and 3% sucrose in a Petri dish or onto soil. Seeds were incubated for 3 days at 4°C to synchronize germination. Plants were grown under light conditions of 100–120 μmol m^−2^ s^−1^ photosynthetic flux.

### RNA isolation and RT-PCR analysis

Roots, stems, leaves from 4-week-old wild type plants and flowers from 5-week-old wild type plants were collected. Ten-day-old seedling plants with or without 5 mM H_2_O_2_ were collected at 0, 1, 3, 6, and 12 h. Ten-day-old seedling plants with or without heat shock treatment at 37°C were collected at 0, 1, 3, 6, 12, and 24 h. These collected samples were frozen with liquid nitrogen for total RNA isolation and cDNA synthesis. Total RNA was extracted from the collected samples using the MACHEREY-NAGEL RNA kit (Düren, Germany) and reverse-transcribed using RevertAid Reverse Transcriptase (Thermo Scientific RevertAid First Strand cDNA Synthesis Kit, Lithuania) according to the manufacturer instructions. The newly synthesize cDNA was diluted to the 50 ng/μL with ddH_2_O. The PCR program initially started with a 95°C denaturation for 2 min, followed by 24 cycles of 95°C/20 s, 60°C/40 s, 72°C/1 min and finally with elongation step at 72°C for 5 min. Specific PCR primers for genes encoding *AtUSP* (At3g53990) were used (AtUSP Forward: 5′-GAATTCCATGCCTAAAGACAGGAATATCGG-3′, AtUSP Reverse: 5′-ATCGATTTATTCGTTATCCTTGACAACGGT-3′). The gene expression levels of *AtUSP* were compared with the internal control gene, *Tubulin* (AT5G62690). Specific PCR primers for genes encoding *Tubulin* (AT5G62690) were used (Tubulin Forward: 5′-CCAACAACGTGAAATCGACA-3′, Tubulin Reverse: 5′-TCTTGGTATTGCTGGTACTC-3′). PCR products were observed in 1% agarose gel electrophoresis stained with ethidium bromide.

### Cloning of *AtUSP* and preparation of transgenic plants over-expressing AtUSP

*AtUSP* was cloned from an *Arabidopsis* cDNA library by the polymerase chain reaction (PCR), as previously described (Bréhelin et al., 2000; Park et al., 2009). After confirmation of the entire coding sequence, the full-length *AtUSP* cDNA was ligated into *EcoRI*/*Cla*I sites of the binary vector pCAMBIA1300, which has a FLAG-tag in the N-terminal region (Figure S1C). *Agrobacterium tumefaciens* GV3101 was transformed with the plasmid and used to transfect *Arabidopsis* by the floral dip method (Clough and Bent, 1998). Transformants were selected on MS plates containing 50 μg/ml hygromycin (Duchefa). AtUSP expression was analyzed using western blot analysis with FLAG-tag antibody (Sigma).

### Hydrophobicity analysis

The ProtScale database (http://www.expasy.org/tools/protscale.html) was used to analyze the hydrophobicity of AtUSP. Hydrophobicity plot of AtUSP was generated by the Kyte–Doolittle analysis (Kyte and Doolittle, 1982).

### Purification of AtUSP recombinant protein

The full-length cDNA of *AtUSP* was isolated from an *Arabidopsis* cDNA library and ligated into the *Bam*HI/*Xho*I sites of the pET28a expression vector (NEB), and the resulting DNA constructs were used to transform *E. coli* BL21 (DE3) cells. The transformants were cultured at 37°C in LB medium containing ampicillin (50 μg/ml) and chloramphenicol (12.5 μg/ml). The culture was diluted 1:50 in LB medium containing 50 μg/ml ampicillin and grown at 30°C until an OD_600_ of 0.6–0.8 was reached. Expression of recombinant protein was induced by the addition of 0.5 mM isopropyl-ß-D-thiogalacto-pyranoside (IPTG) and the cells were grown for a further 4 h. The cells were then harvested by centrifugation at 5000 g for 10 min, and the pellet was resuspended in PBS buffer (140 mM NaCl, 2.7 mM KCl, 10 mM Na_2_HPO_4_, and 1.8 mM KH_2_PO_4_, pH 7.6) with 1 mM PMSF. Cells were stored at −80°C until used. The frozen cells were disrupted by sonication and the soluble extract was loaded onto Ni-NTA agarose columns. Recombinant AtUSP was eluted from the columns by thrombin and dialyzed against 50 mM Hepes-KOH (pH 8.0) at 4°C. The recombinant protein was further purified by FPLC using a Superdex 200 HR 10/30 column. The purity of AtUSP was determined using SDS-PAGE.

### Size exclusion chromatography (SEC)

SEC or FPLC (AKTA; Amersham Biosciences, Uppsala, Sweden) was performed using a Superdex 200 HR 10/30 column from GE Healthcare equilibrated with 50 mM Hepes-KOH (pH 8.0) buffer with a flow rate of 0.5 ml/min at 25°C. Fractions corresponding to protein peaks (A_280_) were isolated and concentrated at 4°C using a Centricon YM-30 filter (Millipore Co., Santa Clara, USA) (Park et al., 2009; Jung et al., 2013).

### Analysis of molecular chaperone activity

Chaperone activity of AtUSP was measured using Malate dehydrogenase (MDH) from Sigma-Aldrich (Missori, USA) as a substrate. MDH was incubated in 50 mM Hepes-KOH (pH 8.0) buffer with various concentrations of AtUSP at 45°C. During the 20 min incubation, thermal aggregation of MDH was determined by monitoring the turbidity at A_340_ using a DU800 spectrophotometer equipped with a thermostatic cell holder, as previously described (Lee et al., 2009; Jung et al., 2013).

### Fluorescence measurement

Fluorescence was measured using a SFM 25 spectrofluorometer (Kontrom, Zurich, Switzerland) was used to measure fluorescence of bis-ANS [1,1-bi(4-anilinonaphthalene-5-sulfonic acid)] obtained from Sigma-Aldrich (Missori, USA). The excitation wavelength of bis-ANS florescence was set to 380 nm, and the emission spectra were scanned between 400 and 600 nm, as described previously (Jung et al., 2013). Reaction mixtures containing 10 μM AtUSP in 50 mM Hepes (pH 8.0) were incubated with 10 μM bis-ANS for 30 min at 25°C.

### Stress treatments and measurements of chlorophyll content and electrolyte leakage

For oxidative stress treatment, leaf discs 7 mm in diameter were collected from the same stages and positions of leaves in wild-type (WT) plants, AtUSP-over-expression lines, and *Atusp* knock-out mutant plants. Discs were immersed abaxial side up in 3 ml of a 0.1% Tween 20 solution containing 10 mM H_2_O_2_. Leaf discs were also subjected to vacuum infiltration for 1 min or incubated at room temperature under light (300 μmol m^−2^ s^−1^) conditions for 18–24 h, and the resulting damage was examined. For heat shock treatment, *Arabidopsis* seedlings were grown on MS media containing 3% sucrose in a Petri dish. Heat shock was applied by placing plates containing 10-day-old *Arabidopsis* seedlings in a water bath at 43°C. Chlorophyll content, extracted using 80% (v/v) acetone, was measured, as described (Porra et al., 1989; Park et al., 2009), and electrolyte leakage was analyzed, as previously reported (Sukumaran and Weiser, 1972; Ristic and Ashworth, 1993). For the measurement of ion conductivity, nine leaf discs (10 mm diameter) were placed in a tube test with 25 ml of de-ionized water and treated with heat shock or oxidative stress with overnight shaking. After autoclaving, the tubes containing leaves were cooled to room temperature and conductivity was measured again. The electrolyte leakage was calculated by the percentages of the conductivity before and after autoclaving. The electrolyte leakage assay was performed at least three times, each time with three replicates.

### Statistical analysis

All values reported in experiments for chlorophyll content, ion leakage, and fresh weight measurements are mean of six replicates. Statistical Analysis were performed using SPSS 12.0.1 software (SPSS Inc., Chicago, IL). One-way ANOVA was used to analyze data (*p* < 0.05) and differences among treatments were performed through Tukey tests.

## Results

### Heat shock- and oxidative stress-dependent expression of *AtUSP* in *Arabidopsis*

**Figure 1:**
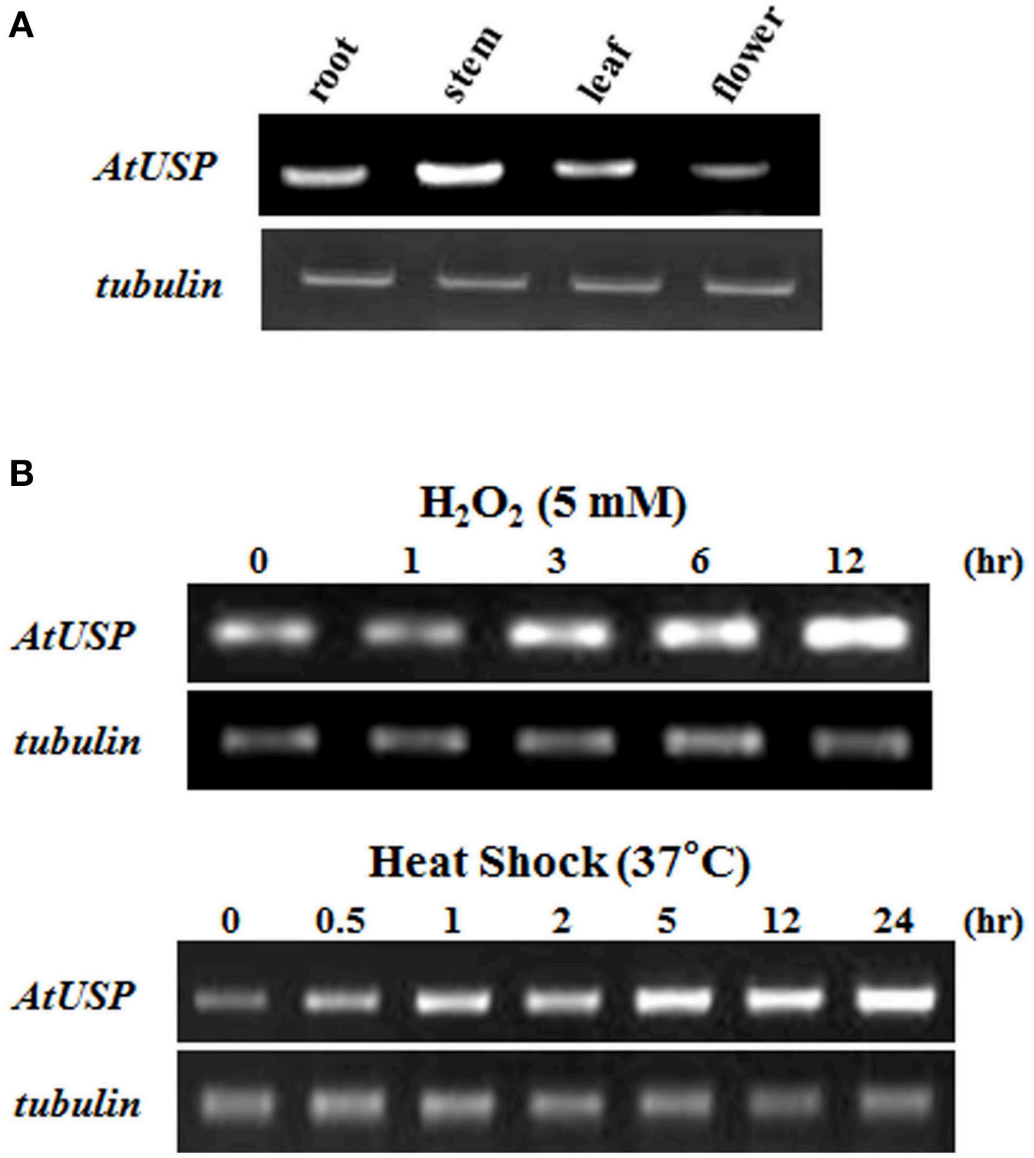
**Expression of ***AtUSP*** mRNA in different tissues and in response to heat shock and oxidative stresses. (A)** Expression of *AtUSP* in various tissues of *Arabidopsis*. **(B)** Expression of *AtUSP* following treatment with 5 mM H_2_O_2_ or heat shock at 37°C. Time represents the treatment hours of plants with H_2_O_2_ and heat shock. *Tubulin* was used as a control.

Even though a number of USPs have been identified as stress-resistance molecules from diverse organisms, the molecular mechanism of USP action has not been resolved (Nyström and Neidhardt, 1992; Zarembinski et al., 1998; Sousa and McKay, 2001; Kvint et al., 2003; Nachin et al., 2005; Persson et al., 2007). Among 44 USP proteins in *Arabidposis*, especially AtUSP (At3g53990) was shown to induce various abiotic stresses in Arabidopsis eFP Browser (http://www.bar.utoronto.ca/) and published papers (Kawamura and Uemura, 2003; Ndimba et al., 2005; Isokpehi et al., 2011). We therefore chose AtUSP (At3g53990) and attempted to unravel the biochemical and molecular functions of a USP associated with stress resistance in *Arabidopsis*. RT-PCR was used to examine the expression levels of *AtUSP* in plants and showed *AtUSP* was widely expressed in most tissues of *Arabidopsis*, including the root, stem, leaf, and flowers (Figure 1A). Since mRNA encoding similar proteins is expressed in various biotic and abiotic stress conditions in other organisms (Nyström and Neidhardt, 1992; Zarembinski et al., 1998; Sousa and McKay, 2001; Kvint et al., 2003; Nachin et al., 2005; Persson et al., 2007), we analyzed expression of *AtUSP* mRNA in *Arabidopsis* after treatment with H_2_O_2_ and heat shock. The transcript levels of *AtUSP* in 10-day-old *Arabidopsis* seedlings gradually increased not only after oxidative stress but also under heat shock conditions (Figure 1B), suggesting that AtUSP plays an important role in the defense system in plant tissues.

### AtUSP enhances resistance of plants to heat shock and oxidative stress

As the expression of *AtUSP* was significantly increased by heat shock or oxidative stress (Figure 1), we investigated its physiological functions using the T-DNA insertion knock-out line of *Atusp* (SALK_146059: Figure S1A) and transgenic plants over-expressing *AtUSP* driven by the CaMV35S promoter (Figure S1B) under stress conditions. PCR analysis of genomic and cDNA expression levels confirmed that *Atusp* is a homozygous loss-of-function mutant of *Arabidopsis* (Figure S1A). In addition, more than 20 independent transgenic lines of plants over-expressing AtUSP were generated. Of the homozygous T3 lines of *Arabidopsis* over-expressing AtUSP, we selected lines #12 and #15 for further studies as these showed the strongest expression of AtUSP when analyzed using a FLAG-tag antibody (Figure S1B).

**Figure 2:**
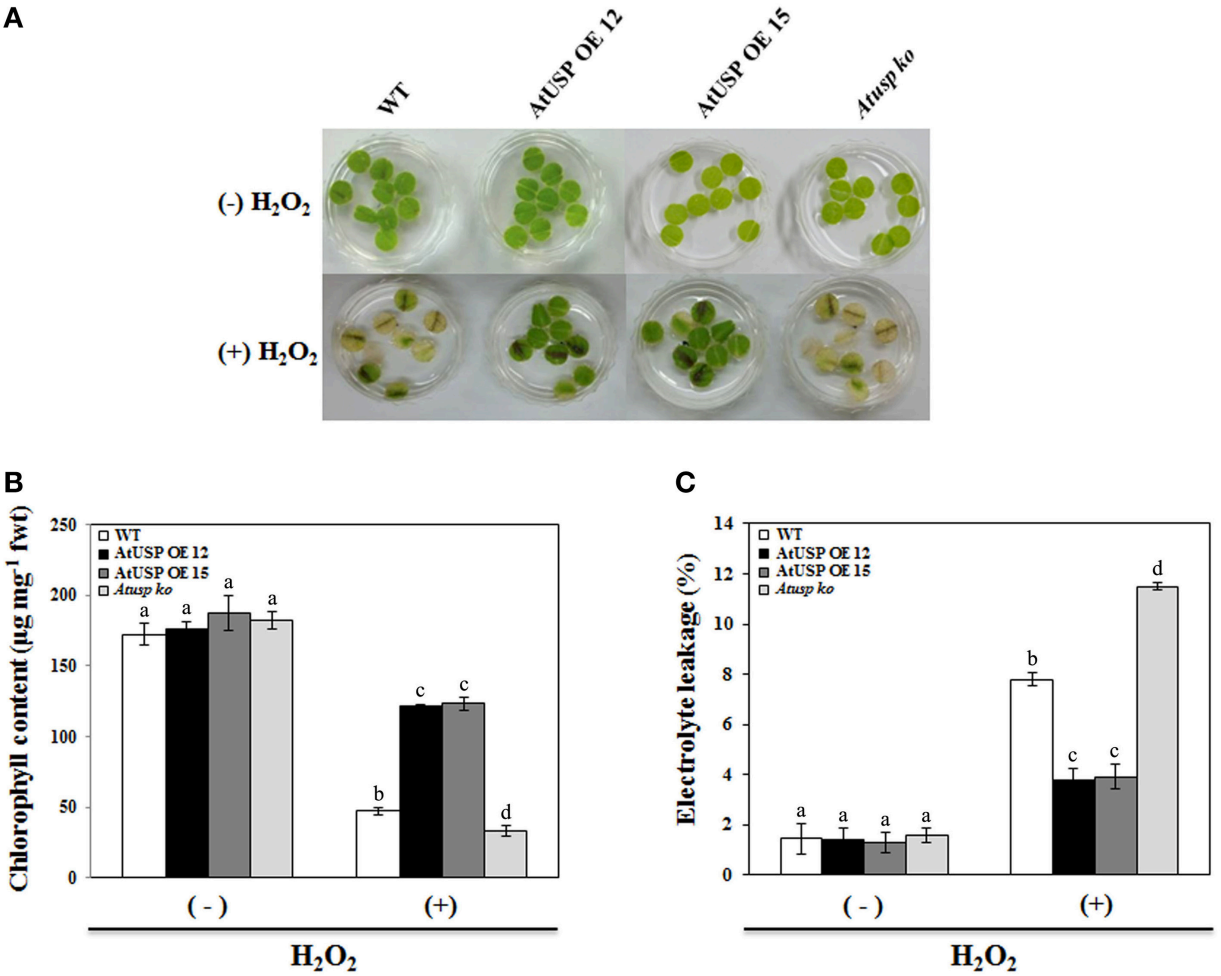
**Comparison of tolerance of oxidative stress in wild-type, AtUSP over-expressing, and ***Atusp*** knock-out mutant ***Arabidopsis*****. **(A)** Nine leaf discs isolated from 3-week-old wild-type, AtUSP over-expressing, and *Atusp* knock-out mutant plants were vacuum infiltrated with water or 10 mM H_2_O_2_ for 1 min and transferred to a growth chamber at 22°C. Samples were collected after 5 days for measurements of chlorophyll content **(B)** and electrolyte leakages **(C)**. All values are means for six replicates ±SE. Data were analyzed using a One-way ANOVA and Tukey test was used to compare the difference between treatments. Different letters indicate the significant differences among the different plant lines (*p* < 0.05).

To examine the *in vivo* role of AtUSP under oxidative stress conditions, leaf discs were collected from 3-week-old *Arabidopsis* seedlings of the WT plants, *Atusp* mutants, and AtUSP over-expression lines, and treated with 10 mM H_2_O_2_. Discs from AtUSP over-expression lines and *Atusp* mutants showed tolerant and sensitive phenotypes compared with those of the WT plants within 5 days of treatment with 10 mM H_2_O_2_, respectively (Figure 2A). Stress tolerance of the over-expression lines and sensitivity of the *Atusp* were confirmed by measuring the total chlorophyll content and electrolyte leakage under stress conditions. The results were consistent with the phenotypic differences (Figure 2A), as discs from plants over-expressing AtUSP showed three-fold higher chlorophyll content and lower electrolyte leakage than discs from WT plants and discs from *Atusp* plants showed about two-fold lower chlorophyll content and higher electrolyte leakage than discs from WT plants under the same conditions (Figures 2B,C).

**Figure 3:**
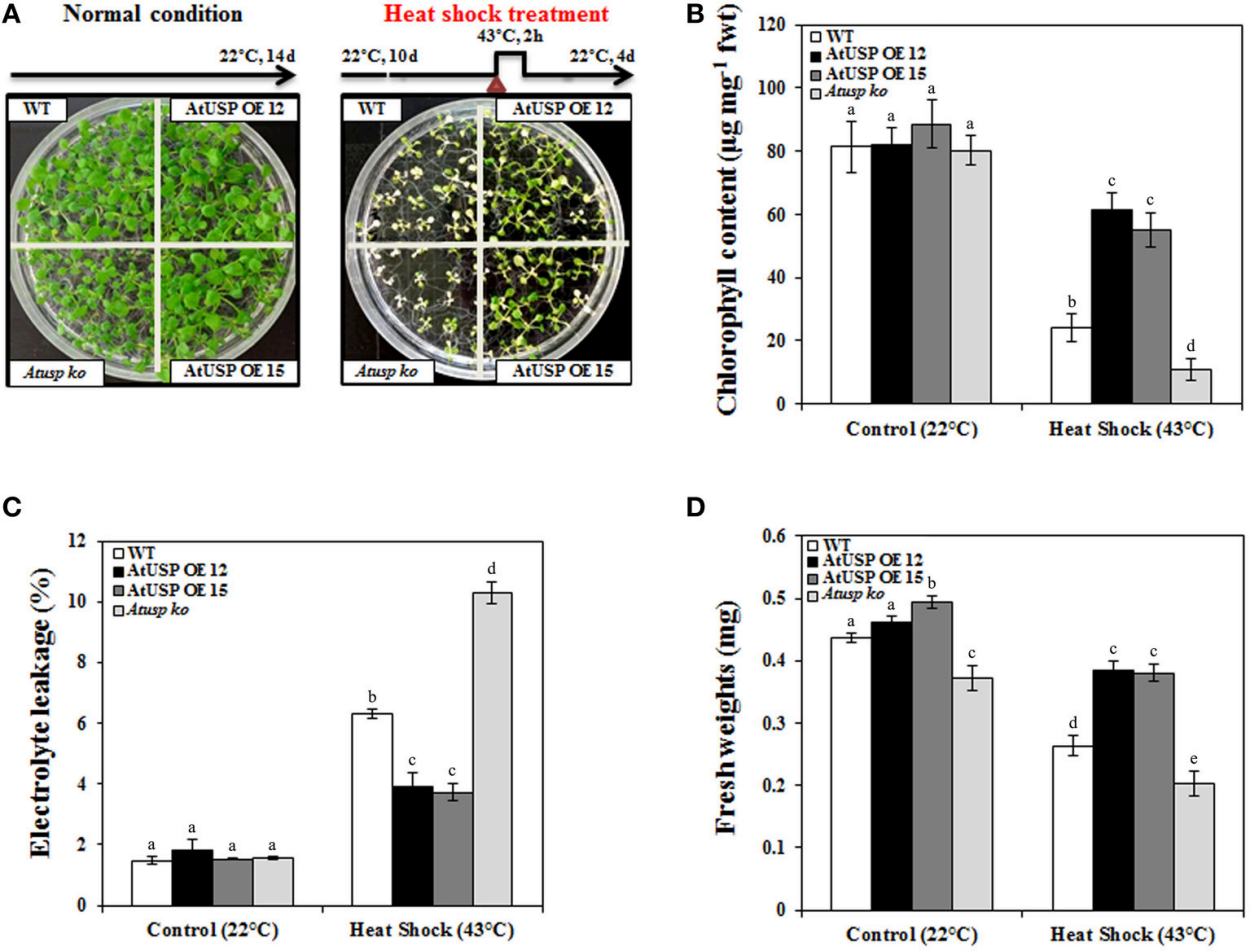
**Comparison of heat shock resistance in wild-type, AtUSP over-expressing, and ***Atusp*** knock-out mutant ***Arabidopsis***. (A)** Ten-day-old *Arabidopsis* seedlings were heat shocked, as indicated, and then transferred to optimal growth conditions at 22°C. Thermo-tolerance of the plants was examined under optimal conditions after a 4 day recovery period. Ten-day-old *Arabidopsis* seedlings were grown at 22°C for 4 days as a control. Chlorophyll (**B**), electrolyte leakage **(C)**, and fresh weight **(D**) were measured. All values are means for six replicates ±SE. Data were analyzed using a One-way ANOVA and Tukey test was used to compare the difference between treatments. Different letters indicate the significant differences among the different plant lines (*p* < 0.05).

In addition to oxidative stress, the physiological significance of AtUSP in *Arabidopsis* under heat shock conditions was examined. A 2 h heat shock (43°C) treatment was applied to 10-day-old *Arabidopsis* seedlings from the WT, *Atusp*, and AtUSP over-expressing lines. Following heat shock treatment, all plants were transferred to their optimal growth conditions at 22°C to allow recovery (upper, Figure 3A). Most plants from the over-expression lines recovered from heat shock compared with the WT plants and *Atusp* mutants, as their growth and development with greenish pigments were restored. Also the *Atusp* mutants showed white or pale leaves and appeared dead than the WT plants indicating that the *Atusp* mutants were unable to recover from heat stress (Figure 3A). Examination of electrolyte leakage, chlorophyll content, and fresh weight also indicated that plants over-expressing AtUSP and *Atusp* mutants were strongly resistant and sensitive to heat shock compared with WT plants, respectively (Figures 3B–D). This suggests that AtUSP plays a very important role in the protection of plants from heat shock and oxidative stress, and is consistent with the data reported for the actions of USPs in microorganisms (Nachin et al., 2005).

### Redox-dependent structural and functional switching of AtUSP in response to oxidative stress

Structural changes have been reported in many kinds of small heat shock proteins (sHSPs) that have a chaperone function and protect cells from heat shock and oxidative stress (Hendrick and Hartl, 1993; Haley et al., 1998). The structural transformation is induced by changes in their hydrophobicity (Park et al., 2009). In particular, several redox proteins, including AtTrx-h3, AtTDX, and 2-Cys peroxiredoxin (2-Cys Prx), which play crucial roles in oxidative and heat shock tolerance and behave as molecular chaperones, show structural changes from low molecular weight (LMW) to high molecular weight (HMW) structures (Jang et al., 2004; Lee et al., 2009; Park et al., 2009).

Because AtUSP was also shown to protect *Arabidopsis* from heat shock and oxidative stress, we investigated its chaperone function and structural changes in response to oxidative stress. First, we analyzed the hydrophobicity of AtUSP using the ProtScale database (http://www.expasy.org/tools/protscale.html). This analysis indicated a structure for AtUSP that contained many hydrophobic regions, which may contribute to its protein stability and polymeric structure (Figure S2). USPs stimulated by various kinds of stresses (Nachin et al., 2005; Loukehaich et al., 2012) are known to be target proteins of thioredoxin (Trx), which regulates the redox status of its interaction partners (Mata-Cabana et al., 2007; Montrichard et al., 2009; Meyer et al., 2012). This observation, together with our finding that AtUSP enhanced the tolerance of plants to oxidative stress, suggested that AtUSP might be a redox-regulated protein.

**Figure 4:**
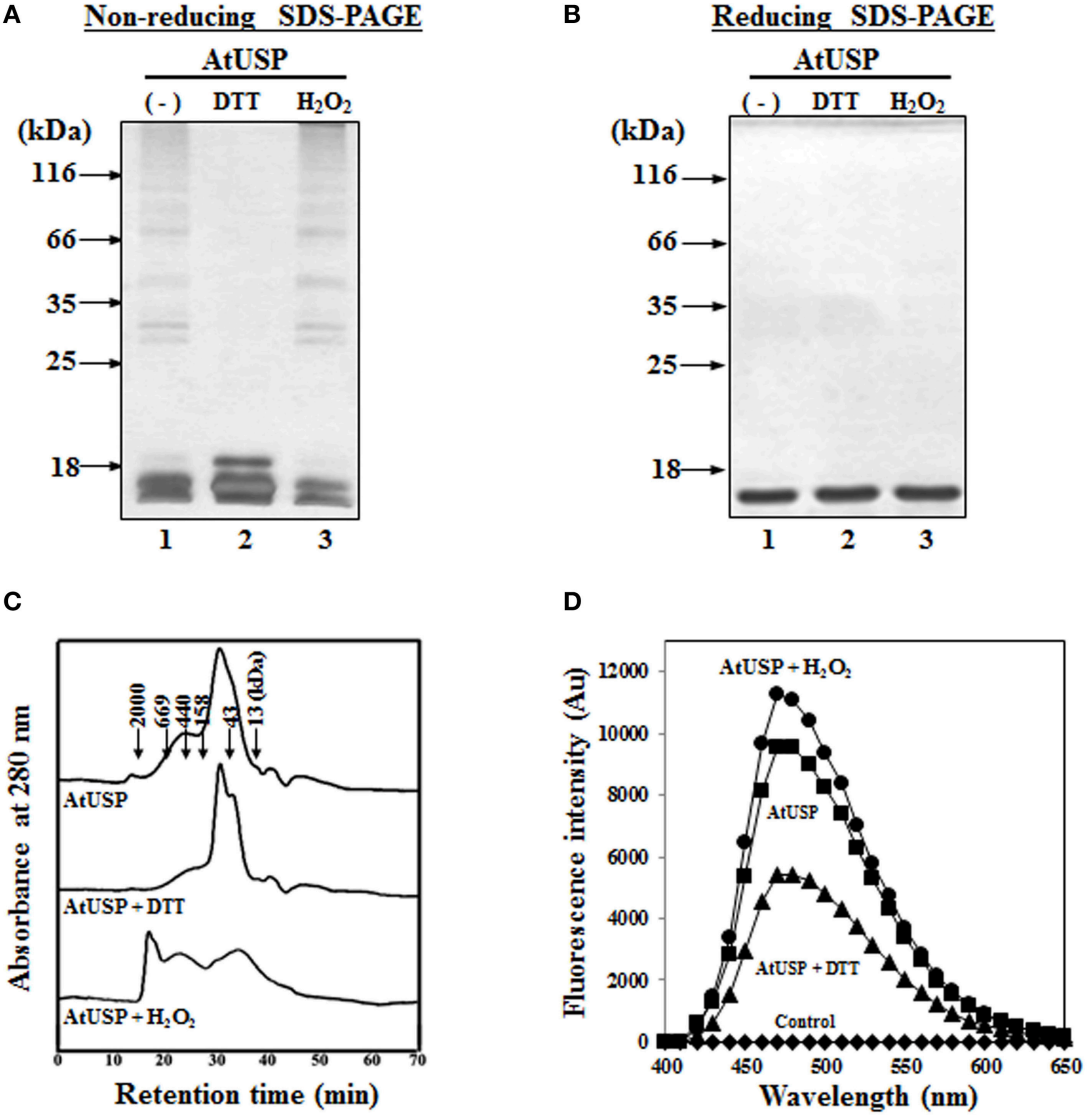
**Redox-dependent structural changes of AtUSP ***in vitro*****. Redox-dependent structural changes of recombinant AtUSP were analyzed by silver staining 12% non-reducing SDS-PAGE **(A)** and reducing SDS-PAGE **(B)** gels. **(A,B)** Purified AtUSP (lane 1) and AtUSP treated with 50 mM DTT (lane 2) were loaded onto the PAGE gels. After removal of DTT by dialysis from the sample used in lane 2, AtUSP was treated with 50 mM H_2_O_2_ (lane 3) and loaded onto the SDS-PAGE gels. **(C)** Redox-dependent structural changes of AtUSP treated the same way as in **(A)** were analyzed using SEC. **(D)** Comparison of the binding affinity of 10 μM bis-ANS to AtUSP (10 μM). Fluorescence intensities of bis-ANS were measured using an excitation wavelength of 390 nm and emission wavelengths from 400 to 600 nm. (♦) bis-ANS alone, (■) untreated AtUSP, (▴) AtUSP treated with DTT, and (•) AtUSP treated with H_2_O_2_.

To determine whether, like several redox-related proteins, such as Trx and Prx, AtUSP exhibited redox-dependent structural changes *in vitro*, we expressed recombinant AtUSP in bacteria. The recombinant AtUSP was purified and then its redox-dependent structural changes were tested under reducing and non-reducing SDS-PAGE conditions. AtUSP was sequentially treated with DTT and H_2_O_2_, electrophoresed using PAGE, and examined for structural changes. Under non-reducing conditions, the protein structure of AtUSP consisted of several oligomeric protein complexes with the monomeric protein as a major band (Figure 4A, lane 1). However, treatment with 10 mM DTT caused the oligomeric structures of AtUSP to dissociate into the monomer (Figure 4A, lane 2). This suggested that the homo-polymeric structure of AtUSP was produced by linking a varying number of AtUSP subunits with disulfide bonds.

To determine whether this change in AtUSP protein structure showed redox-dependent reversibility, DTT was completely removed from AtUSP by dialysis and then the protein was treated with 10 mM H_2_O_2_ for 30 min to ensure that AtUSP, which had been monomerized by DTT treatment, was repolymerized by the oxidizing agent, H_2_O_2_. The treated samples were analyzed using non-reducing SDS-PAGE. The result indicated that the reduced thiol groups of AtUSP had been reoxidized to form disulfide bonds and polymeric structures (Figure 4A, lane 3). By contrast, under reducing SDS-PAGE conditions, AtUSP displayed as a single protein band with a MW was estimated at 17.8 kDa, suggesting that treatment with β-mercaptoethanol resulted in the complete dissociation of multimeric AtUSP into its monomers (Figure 4B). The redox-dependent structural changes of AtUSP were independently confirmed using SEC. The protein structure of AtUSP was reversibly shifted from LMW species to HMW complexes by treatment with DTT or H_2_O_2_ (Figure 4C). Next, binding of bis-ANS, a measure of structural changes was determined following treatment of AtUSP with DTT and H_2_O_2_. The fluorescence intensity of H_2_O_2_-treated AtUSP was significantly higher than that of untreated or DTT-treated AtUSP (Figure 4D).

**Figure 5:**
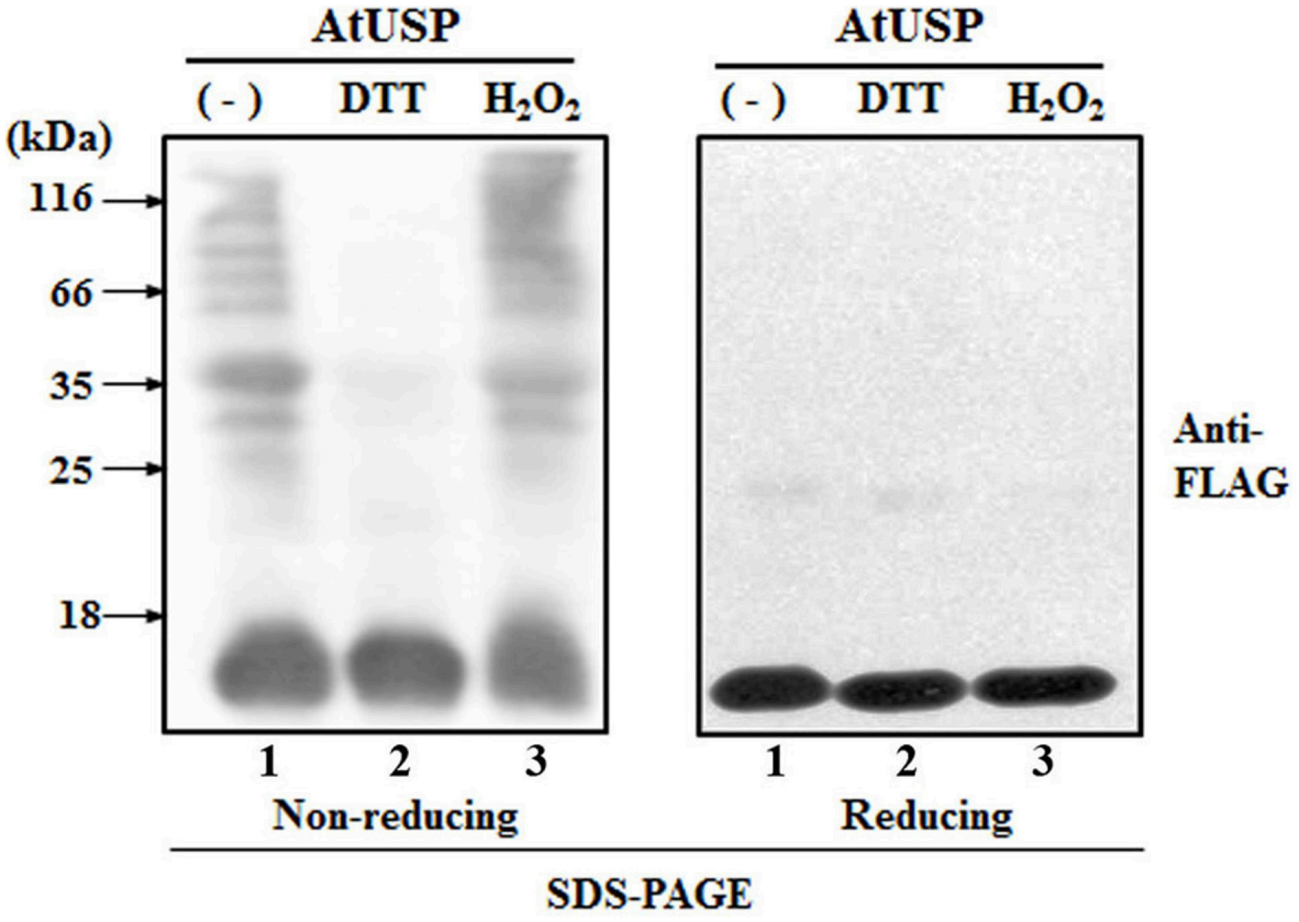
**Redox-dependent structural changes of AtUSP ***in vivo*****. Samples from 2-week-old transgenic plants over-expressing AtUSP fused to FLAG were prepared and treated with 10 mM H_2_O_2_ and DTT. The proteins were loaded onto non-reducing (left-hand) and reducing (right-hand) SDS-PAGE gels. Protein structures were determined by western blotting using FLAG-tag antibody. Lane 1: Total protein (30 μg) obtained from an AtUSP over-expression line. Lane 2: Protein sample from an AtUSP over-expression line treated with 10 mM DTT. Lane 3: Protein sample from an AtUSP over-expression line as in lane 2 after removal of DTT by dialysis and treatment with 10 mM H_2_O_2_.

The structural changes analyzed using *in vitro* experiments were confirmed *in vivo* with studies of 2-week-old transgenic *Arabidopsis* plants from over-expression line #12. Proteins were extracted from plants treated with DTT or H_2_O_2_ by vacuum infiltration, and subjected to SDS-PAGE under reducing and non-reducing conditions. Under non-reducing conditions, *in vivo* AtUSP showed multiple protein bands, consisting of a number of oligomeric proteins and a monomeric band, despite the presence of an excessive amount of SDS (1%) (Figure 5, lane 1). This indicated that the major factor in oligomerization was the hydrophobic interaction. When, however, AtUSP was incubated with DTT prior to analysis in non-reducing conditions, the oligomeric structures of AtUSP were almost completely dissociated into monomers (Figure 5, lane 2), although treatment of the monomeric form with H_2_O_2_ restored the native polymeric structures (Figure 5, lane 3). Taken together, the *in vitro* and *in vivo* results indicate that individual proteins of AtUSP can form polymeric complexes through hydrophobic interactions and disulfide bonds, and the formation of these structures is reversibly regulated in a redox-dependent manner.

### AtUSP exhibits a chaperone function

It is well-known that molecular chaperones not only prevent aggregation of nascent proteins in cells but also facilitate their correct folding by protecting substrate aggregations from stresses (Li et al., 2013). Chaperone proteins exist as multimeric conformations consisting of dimers, trimers, and higher oligomeric complexes (Haley et al., 1998); AtUSP was also shown to form polymeric structures, so we examined whether it had a chaperone function. MDH was used as a substrate to assess the ability of AtUSP to inhibit thermal aggregation of proteins; AtTrx-h3 was a positive control.

**Figure 6:**
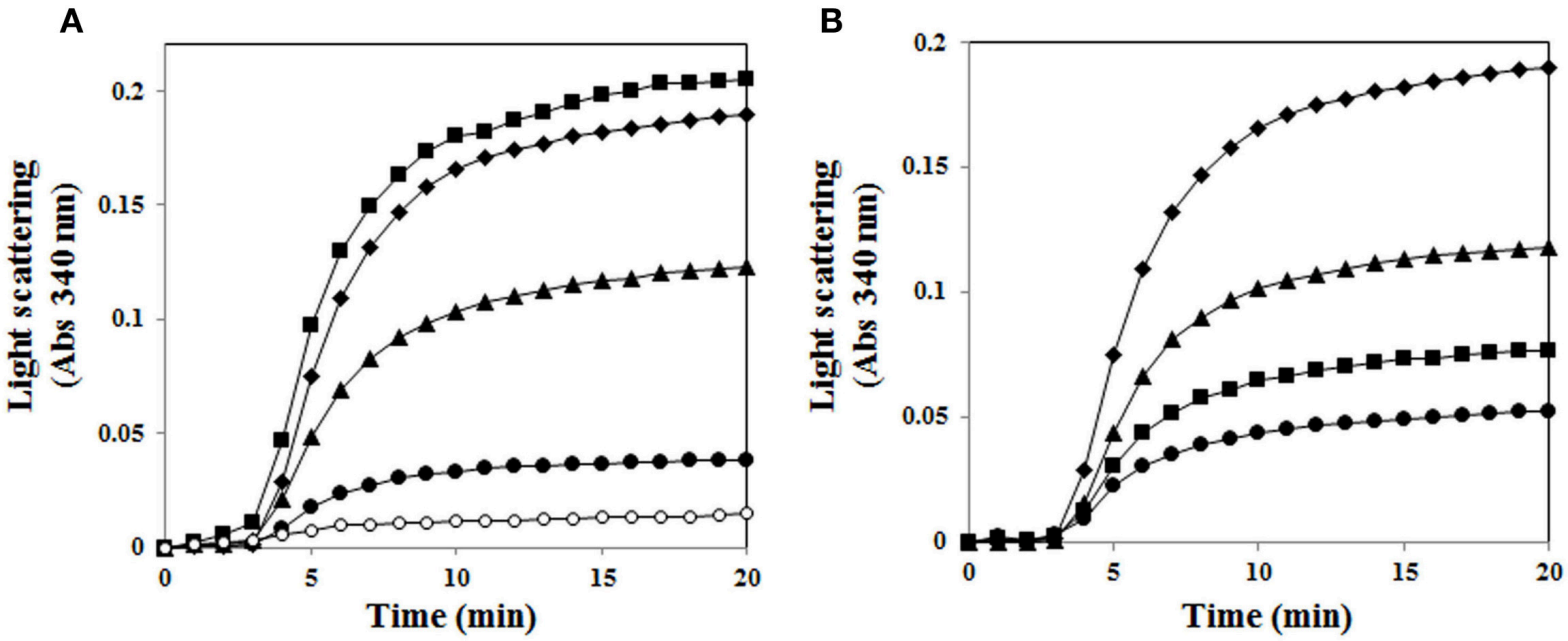
**Redox-dependent chaperone function of AtUSP. (A)** Thermal aggregation of 1.5 μM MDH was examined at 45°C for 20 min in the presence of AtUSP. Molar ratios of AtUSP to MDH were (■) 1:0.5, (▴) 1:1, and (•) 1:3. (♦) Negative control (MDH alone), (°) Positive control (AtTrx-h3). **(B)** Redox-dependent chaperone activity of AtUSP with MDH. (♦) MDH alone, (■) AtUSP, (▴) DTT-treated AtUSP, and (•) H_2_O_2_-treated AtUSP were used to measure the level of protection against thermal aggregation of MDH.

Incubation of MDH with an increasing amount of AtUSP gradually prevented the thermal aggregation of substrate, which was significantly blocked at a subunit molar ratio of 1 MDH to 3 AtUSP (Figure 6A). This indicates AtUSP is a novel molecular chaperone that can protect plants from diverse external stresses. In addition, because AtUSP protein structure was reversibly regulated by its redox status, we investigated whether its chaperone function was redox dependent. Chaperone activity was measured using AtUSP treated with DTT or H_2_O_2_. H_2_O_2_-treated AtUSP showed much stronger chaperone activity in protecting MDH from aggregation than protein treated with DTT, suggesting that AtUSP is an efficient molecular chaperone whose activity is regulated in a redox-dependent manner (Figure 6B).

### Effect of heat shock on the protein structure and chaperone function of AtUSP

**Figure 7:**
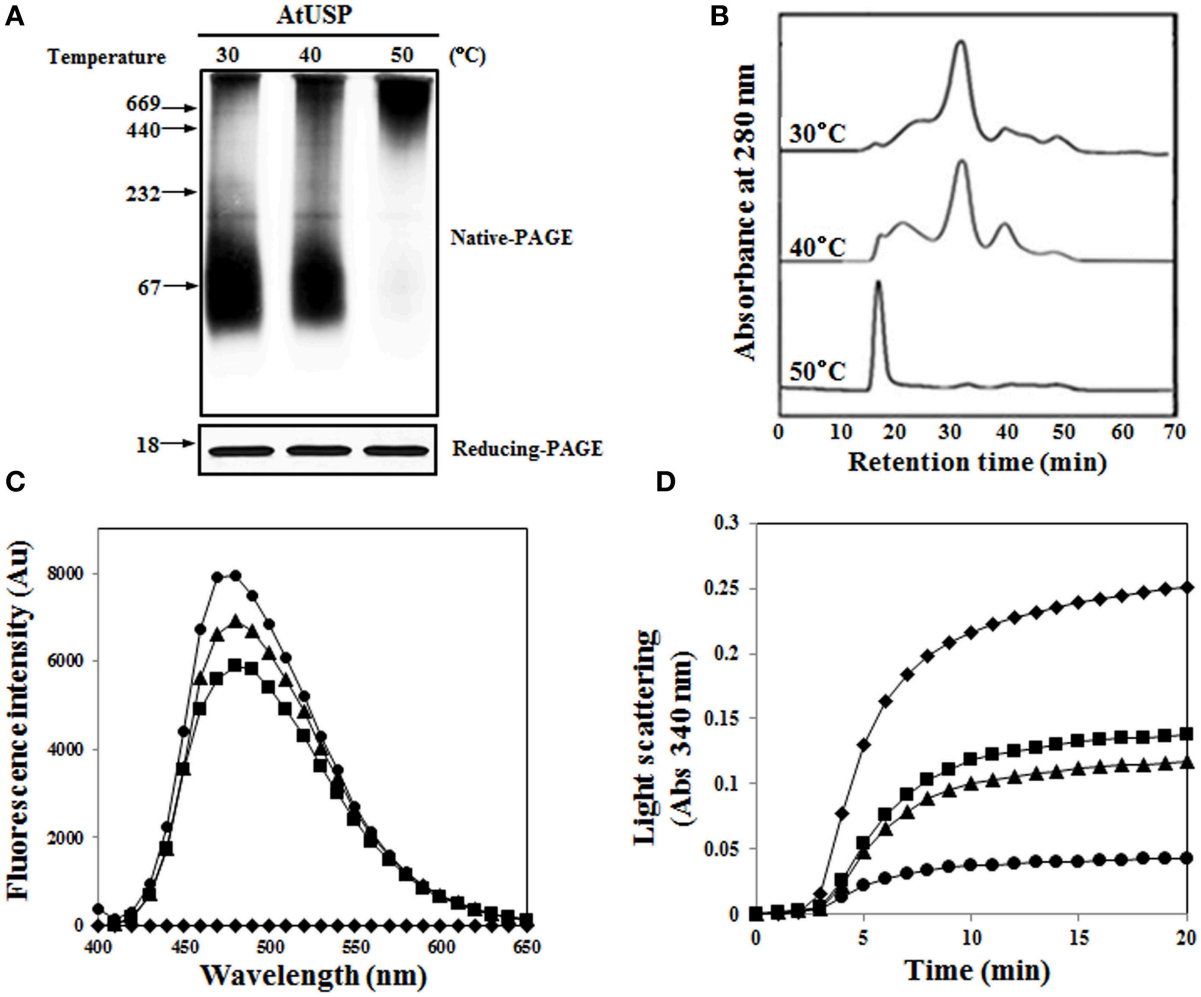
**Effect of heat shock on structural changes of AtUSP. (A)** Recombinant AtUSP was treated with heat shocks at various temperatures for 20 min and the resulting proteins were separated using native-PAGE (upper) and SDS-PAGE gels (bottom). **(B)** AtUSP treated with heat shock was analyzed using SEC to determine structural changes. **(C)** Heat shock-mediated hydrophobicity changes of AtUSP were measured by bis-ANS binding. AtUSP was incubated at (■) 30°C, (▴) 40°C, or (•) 50°C for 30 min. (♦) Control (bis-ANS alone). **(D)** Heat shock-mediated enhancement of the chaperone function of AtUSP. Thermal aggregation of MDH was examined at 45°C for 20 min in the presence of heat shock-treated AtUSP proteins at (■) 30°C, (▴) 40°C, or (•) 50°C for 20 min. (♦) Control (MDH).

Since AtUSP exhibited a molecular chaperone function, we analyzed the effect of heat shock on its protein structure using native-PAGE and SEC. Like many other heat shock proteins (HSPs) (Haley et al., 1998), AtUSP associated to form HMW homo-polymeric complexes under heat shock conditions. Structural changes to AtUSP commenced at around 40°C, and almost all proteins associated into HMW oligomeric complexes following a heat shock treatment at 50°C for 20 min (Figure 7A). This suggested that heat shock caused LMW protein complexes to assemble into HMW complexes. The heat shock-mediated structural changes of AtUSP were confirmed using SEC. As the temperature increased, so did the oligomer peaks of AtUSP, and these changes occurred simultaneously with a decrease in the levels of LMW proteins (Figure 7B). As a measurement of hydrophobicity changes, binding of bis-ANS was analyzed in heat shock-treated AtUSP. The fluorescence intensity of bis-ANS significantly increased at higher temperatures, which indicated that a greater number of hydrophobic regions of AtUSP were exposed at elevated temperatures (Figure 7C). In addition, we measured heat shock-dependent chaperone activity of AtUSP, using MDH as a substrate. The chaperone activity of AtUSP was significantly enhanced by increasing the incubation temperature of AtUSP (Figure 7D). All these results were consistent with the idea that AtUSP plays a critical role in protecting plants from heat shock and oxidative stresses, and its function as a molecular chaperone is accompanied by reversible changes to its protein structure.

## Discussion

Although the physiological significance of USPs has been well-studied in many organisms, especially in *E. coli*, and expression of USPs is known to respond to various environmental stresses, including salt, drought, cold, heat, oxidative stress, nutrient starvation, and toxic chemicals (Guan and Nothnagel, 2004; Ndimba et al., 2005), the molecular mechanism of USP action has not been identified. USPs are either small polypeptides (14–15 kDa) containing a single USP domain or larger proteins consisting of one or two USP domains together with another functional domain, such as a kinase, permease, or voltage channel domain (Isokpehi et al., 2011). The *Arabidopsis* genome contains 44 USPs that show similarity to the bacterial USP domain and are induced by a variety of stresses (Isokpehi et al., 2011; Loukehaich et al., 2012). AtUSP also shows similarity to genes responsive to ethylene, a plant hormone involved in fruit ripening (Kerk et al., 2003). These observations prompted us to investigate the biochemical functions of USPs in protecting plants from external stresses.

A comparison of the amino acid sequence of AtUSP with USP sequences from other organisms showed a high sequence homology and secondary structural similarity. The USP from *M. jannaschii*, designated as MJ0577, contains five beta strands alternating with four alpha helices (Zarembinski et al., 1998) and a conserved ATP-binding motif, G-2X-G-9X-G-(S/T) (Figure S3), in which three glycine residues are separated by two and nine amino acid residues, followed by a serine or threonine residue. This motif is a typical feature of the USP family (Pfam accession number PF00582) (Kim et al., 2012). Since oxidative stress plays a critical role in all aerobic organisms, the oxidative-associated function of AtUSP may provide much information for understanding the physiological significance of this protein in plant cells (Mayer, 2012).

We identified a novel function for AtUSP, showing that it acted as a molecular chaperone under heat shock and oxidative stress conditions, and that this action was accompanied by a switch in protein structure. Both the chaperone function and the changes in protein structure were regulated in a redox-dependent manner. Under normal conditions, AtUSP protein was present in multimeric complexes but treatment with DTT caused these to dissociate completely into monomers; the polymeric structures could be reversibly restored by H_2_O_2_ treatment. Similarly, the protein conformation of NPR1, a pathogen-responsive defense regulator in plants, also changes from an oligomeric structure to a monomer in the presence of a redox protein (Trx) and pathogen-induced production of salicylic acid (Tada et al., 2008). These structural changes also induced translocation of NPR1 from the cytosol to the nucleus, thus activating defense-related downstream genes. 2-Cys Prx, which also plays dual roles, acting both as a signaling molecule and as a molecular chaperone, likewise shows changes to its protein structure and function according to oxidative stress and redox status. 2-cys Prx can protect many important intracellular proteins under conditions of oxidative stress (Chuang et al., 2006).

The cysteine residues in the AtTrx-h3, 2-Cys Prx, and NPR1 proteins play an important role in regulation by redox status (Jang et al., 2004; Tada et al., 2008; Park et al., 2009). As USP is one of the targets of Trx in plants (Montrichard et al., 2009), the two cysteine residues of AtUSP may also play a critical role in redox regulation, and this should be investigated in the future. We clearly demonstrated the chaperone function of AtUSP using bacterially expressed recombinant protein with MDH as a substrate. The chaperone function of AtUSP was significantly increased under stress conditions by inducing the formation of HMW complexes. This regulation resembles that of AtTrx-h3 and AtNTRC (Park et al., 2009; Chae et al., 2013). We propose that the molecular switch in AtUSP structure results from its ATP-binding motif forming hydrogen bonds with polar groups of amino acids responsible for the oligomerization and higher hydrophobic interaction of AtUSP (Isokpehi et al., 2011).

The significance of chaperone function of AtUSP in plants was verified in transgenic plants over-expressing AtUSP. These plants showed significantly increased tolerance to heat shock and oxidative stress, whereas the knock-out mutant, *Atusp*, showed a sensitive-to-stress phenotype with lower chlorophyll content and higher electrolytic leakage compared to the wild type plants under heat shock and oxidative stress. Under normal conditions, however, there were some differences in growth between *Atusp*, WT plants, and AtUSP over-expression lines, suggesting AtUSP may play another role in plant growth and development. In tomato plants, the *SpUSP* transcript is regulated by a number of phytohormones, such as abscisic acid, gibberellin, or ethylene (Loukehaich et al., 2012), and the expression of rice *USP* is also regulated by ethylene; it is probable, therefore, that *AtUSP* expression is also regulated by plant hormones. This study demonstrates novel physiological and molecular functions of AtUSP, showing that it acts to protect plants from heat shock and oxidative stress. Our observation that AtUSP has a redox-dependent chaperone function expands its known roles in plants and thus increases our understanding of the molecular mechanisms underlying its diverse defensive roles.

## Author contributions

YC, DY, and SL designed the experiments and wrote the paper. YJ, SM, EL, JP, CA, HO, and YC performed the experiments and analyzed the data.

### Conflict of interest statement

The authors declare that the research was conducted in the absence of any commercial or financial relationships that could be construed as a potential conflict of interest.
