# Supplementary material for: Universal Stress Protein Exhibits a Redox-Dependent Chaperone Function in Arabidopsis and Enhances Plant Tolerance to Heat Shock and Oxidative Stress
Source: Front Plant Sci. 2015 Dec 21;6:1141. doi: 10.3389/fpls.2015.01141 (PMC4685093; doi:10.3389/fpls.2015.01141)

Figure S1. Schematic representation and selection of T-DNA knock-out and over-expression lines of AtUSP. (A) *AtUSP* (At3g53990) gene structure showing T-DNA insertion, genotyping, and RT-PCR analysis of *AtUSP* transcript from the SALK_146059 line. **(B)** Construct and selection of AtUSP over-expression lines. Expression of AtUSP was detected by western blotting using a FLAG-tag antibody. Transgenic lines #12 and #15 (marked with asterisks) were selected for further experiments. (C) Nucleotide and amino acid sequence of the construction with 3xFlag fused AtUSP. Red and blue color represents the nucleotide and amino acid sequences of Flag and full-length AtUSP respectively. Black underline indicates Multiple Cloning Site (MCS) region.

Figure S2. Hydrophobicity measurement of AtUSP. A hydrophobicity plot of AtUSP was generated by Kyte-Doolittle analysis. The red line represents the separation of the score of the important hydrophobic residues of AtUSP. The y-axis indicates the hydrophobicity score. Positive scores on the y-axis indicate hydrophobic regions.

Figure S3. Nucleotides and amino acid sequences of AtUSP and multiple sequence alignment (MSA) of USPs from different species. (A) The nucleotide sequence of AtUSP is numbered starting at the first nucleotide of the insert. Amino acid numbering starts at the first methionine residue. The stop codon is marked with an asterisk. The two cysteine residues of AtUSP are outlined in the red boxes. The G2XG9XG(S/T) motif containing putative ATP-binding residues is underlined. **(B)** MSA of USPs from *Arabidopsis* (AtUSP, NP_566991.1), rice (OsUSP1, AAR87267), tomato (SpUSP, SGN-U214690), *Escherichia coli* (EcUspA, U00039), and *M. jannaschii* (MJ0577, NP_247556). The MSA was produced using ClustalW. Conserved amino acids in the sequence alignment are shaded. Amino acids with over 75% conservation are shaded in black; amino acids with between 65% and 75% conservation are shaded in gray. The black bars below the sequences represent residues facing the adenine of ATP (A), the phosphate of ATP (P), or the ribose of ATP (R), or else located in the dimerization domain (D), as derived from the crystal structure of *M. jannaschii* MJ0577. The secondary structural elements of AtUSP were predicted using the Jpred program. The secondary structure of the MJ0577 protein based on its crystal structure is shown above the alignment [E, extended conformation (β-strand); H, α-helix]. Gaps in the alignment are indicated by dashes. Str, Structure.

FIGURE S1

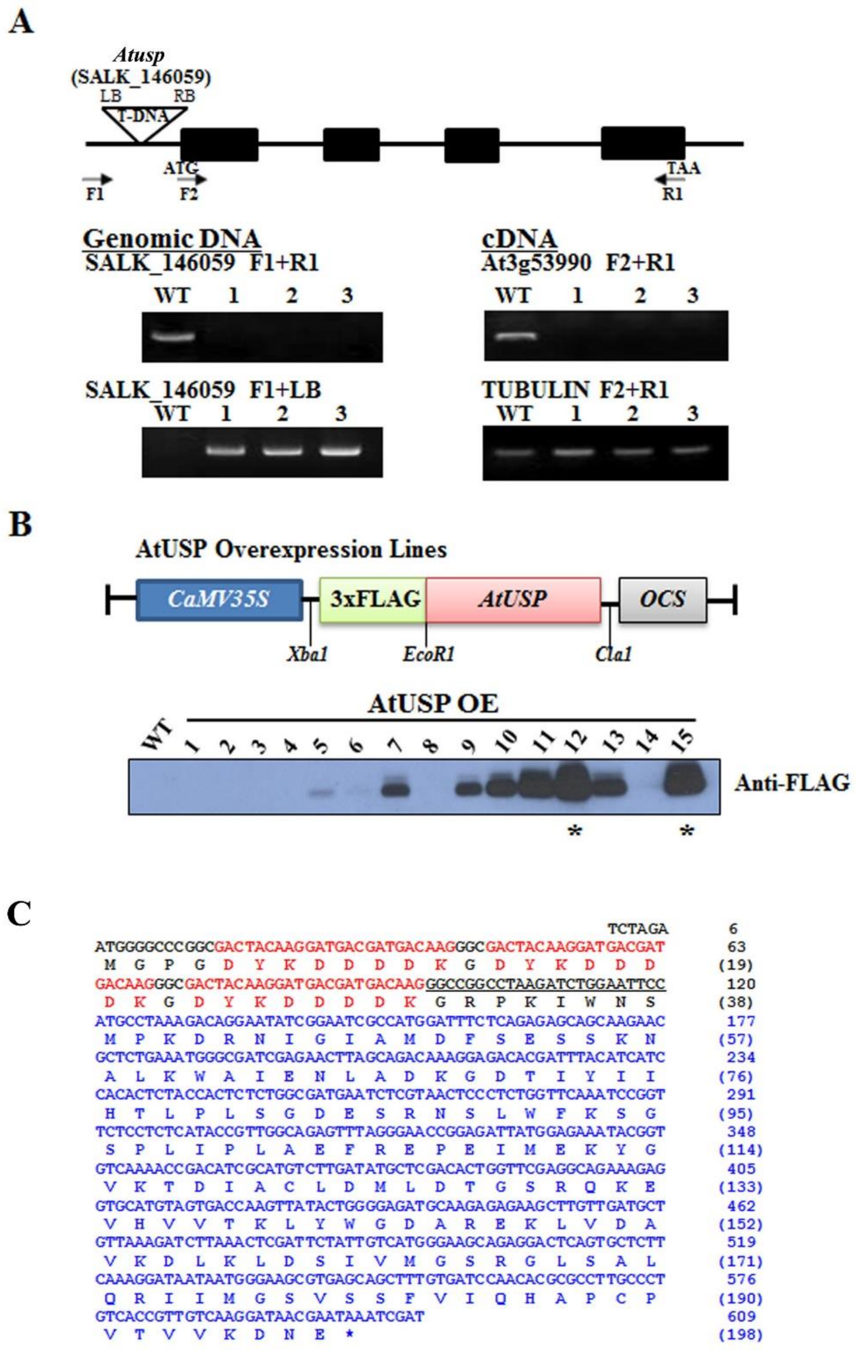

A
Atusp
(SALK_146059)
LB
RB
T-DNA
ATG
TAA
F1
F2
R1
Genomic DNA
SALK_146059 F1+R1
WT 1 2 3
SALK_146059 F1+LB
WT 1 2 3
cDNA
At3g53990 F2+R1
WT 1 2 3
TUBULIN F2+R1
WT 1 2 3
B
AtUSP Overexpression Lines
CaMV35S
3xFLAG
AtUSP
OCS
Xba1
EcoR1
Cla1
AtUSP OE
WT 1 2 3 4 5 6 7 8 9 10 11 12 13 14 15
Anti-FLAG
* *
C
TCTAGA 6
ATGGGGCCCGGCGACTACAAGGATGACGATGACAAGGGCGACTACAAGGATGACGAT 63
M G P G D Y K D D D D K G D Y K D D D (19)
GACAAGGGCGACTACAAGGATGACGATGACAAGGGCCGGCCTAAGATCTGGAATTCC 120
D K G D Y K D D D D K G R P K I W N S (38)
ATGCCTAAAGACAGGAATATCGGAATCGCCATGGATTTCTCAGAGAGCAGCAAGAAC 177
M P K D R N I G I A M D F S E S S K N (57)
GCTCTGAAATGGGCGATCGAGAACTTAGCAGACAAAGGAGACACGATTTACATCATC 234
A L K W A I E N L A D K G D T I Y I I (76)
CACACTCTACCACTCTCTGGCGATGAATCTCGTAACTCCCTCTGGTTCAAATCCGGT 291
H T L P L S G D E S R N S L W F K S G (95)
TCTCCTCTCATACCGTTGGCAGAGTTTAGGGAACCGGAGATTATGGAGAAATACGGT 348
S P L I P L A E F R E P E I M E K Y G (114)
GTCAAAACCGACATCGCATGTCTTGATATGCTCGACACTGGTTCGAGGCAGAAAGAG 405
V K T D I A C L D M L D T G S R Q K E (133)
GTGCATGTAGTGACCAAGTTATACTGGGGAGATGCAAGAGAGAAGCTTGTTGATGCT 462
V H V V T K L Y W G D A R E K L V D A (152)
GTTAAAGATCTTAAACTCGATTCTATTGTCATGGGAAGCAGAGGACTCAGTGCTCTT 519
V K D L K L D S I V M G S R G L S A L (171)
CAAAGGATAATAATGGGAAGCGTGAGCAGCTTTGTGATCCAACACGCGCCTTGCCCT 576
Q R I I M G S V S S F V I Q H A P C P (190)
GTCACCGTTGTCAAGGATAACGAATAAATCGAT 609
V T V V K D N E * (198)

FIGURE S2

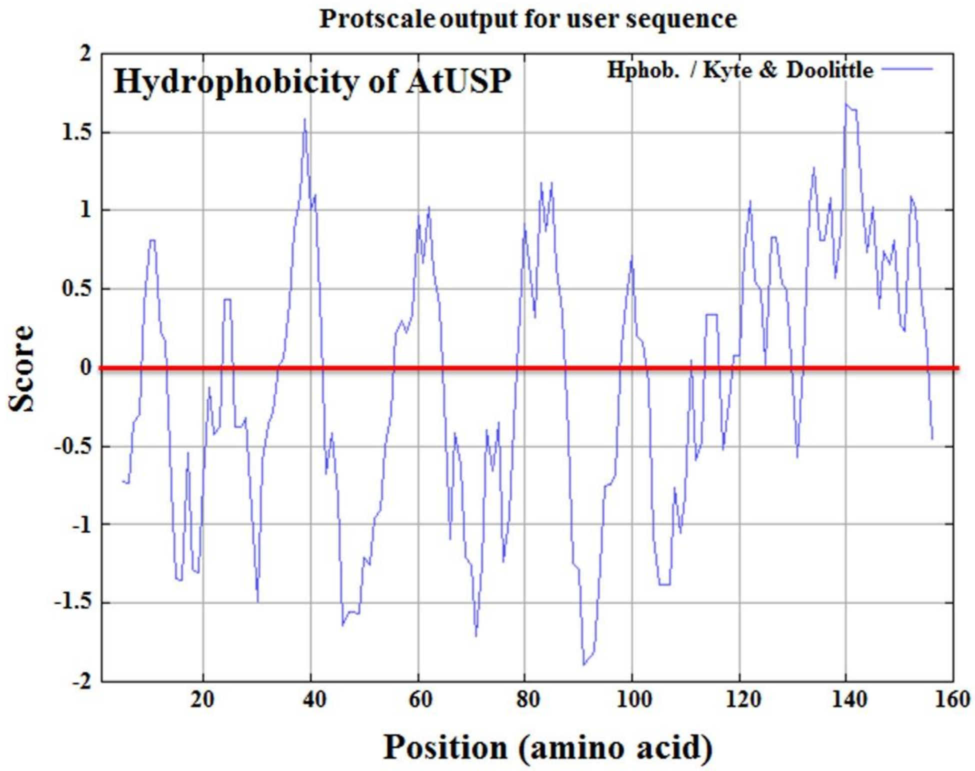
Protscale output for user sequence
Hydrophobicity of AtUSP
Hphob. / Kyte & Doolittle
Score
2
1.5
1
0.5
0
-0.5
-1
-1.5
-2
20
40
60
80
100
120
140
160
Position (amino acid)

FIGURE S3

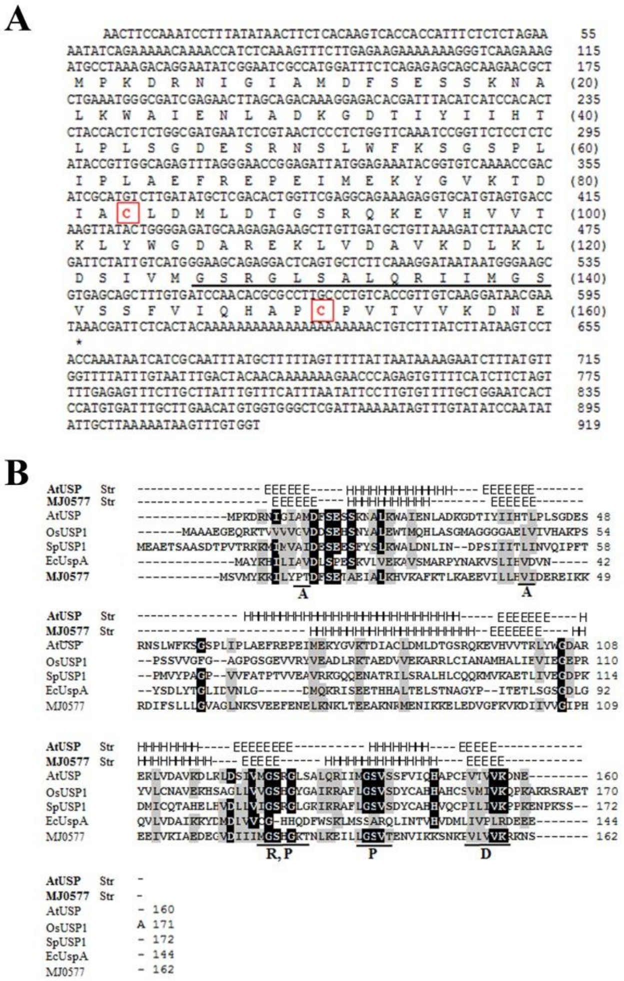

Supplement: Supplementary file 1 [file Presentation1.PDF]
